# Prenatal Selective Serotonin Reuptake Inhibitor Exposure and Its Impact on Neonatal Gastrointestinal and Urinary System: A Retrospective Matched Cohort Study

**DOI:** 10.3390/children13050630

**Published:** 2026-04-30

**Authors:** Ronella Marom, Laurence Mangel, Addy S. BrandStetter, Jacky Herzlich, Dror Mandel, Yuval Bar-Yosef

**Affiliations:** 1 Department of Neonatology, Dana Dwek Children’s Hospital, Tel Aviv Sourasky University Medical Center, Tel Aviv 6423906, Israel; laurencem@tlvmc.gov.il (L.M.);; 2 Gray Faculty of Medical & Health Sciences, Tel Aviv University, Tel Aviv 6997801, Israel; 3 Department of Pediatric Urology, Dana-Dwek Children’s Hospital, Tel Aviv Medical Center, Tel Aviv 6423906, Israel

**Keywords:** selective serotonin reuptake inhibitor, poor neonatal adaptation syndrome, neonates, gastrointestinal system, urinary system

## Abstract

**Highlights:**

**What are the main findings?**

- In a retrospective matched cohort study, in utero SSRI exposure was associated with shorter time to first stool.
- Time to first void did not differ between SSRI-exposed and unexposed neonates.

**What are the implications of the main findings?**

- These findings support a potential serotonergic influence on early neonatal gastrointestinal function.
- The absence of differences in voiding timing is reassuring regarding early neonatal renal–urinary adaptation.

**Abstract:**

Objective: Prenatal exposure to selective serotonin reuptake inhibitors (SSRIs) has been associated with altered neonatal adaptation, but its relationship with early elimination patterns remains unclear. Given the role of serotonin in gastrointestinal and urinary physiology, we aimed to evaluate the association between maternal SSRI use during pregnancy and time to first stool and time to first void in healthy neonates. Methods: In this retrospective matched cohort study, neonates exposed to SSRIs in utero were matched 1:1 with unexposed controls by gestational age (GA) and weight-for-gestational-age category. The primary outcomes were time to first void and time to first stool. Multivariable linear regression was performed using log10-transformed time to first stool, adjusting for maternal age, GA, and neonatal sex. Sensitivity analyses included size-for-gestational-age and time to first feeding. Results: A total of 266 neonates were included (133 SSRI-exposed, 133 unexposed). Time to first stool was shorter in SSRI-exposed neonates compared with unexposed neonates (median 7.4 vs. 8.6 h, *p* = 0.023), while the time to first void did not differ. In adjusted analysis, SSRI exposure remained associated with shorter time to first stool (β = −0.08, 95% CI −0.16 to −0.001, *p* = 0.035), corresponding to an approximate 17% reduction. The association was consistent across sensitivity analyses. Meconium-stained amniotic fluid was associated with shorter time to first stool among SSRI-exposed neonates but not in unexposed neonates. The overall model explained a limited proportion of variance. Conclusions Prenatal SSRI exposure was associated with modest but consistent reduction in time to first stool, without affecting time to first void. While the clinical significance remains uncertain, these findings suggest a potential influence of in utero SSRI exposure on early neonatal gastrointestinal adaptation, which may be influenced by intrapartum conditions.

## 1. Introduction

The use of antidepressant drugs during pregnancy, particularly selective serotonin reuptake inhibitors (SSRIs), has increased over the past few years and is currently estimated at 2–6% of pregnant women [1,2,3,4]. Given their ability to cross the placenta, SSRIs may influence fetal development and neonatal adaptation. Approximately 30% of infants exposed to SSRIs in utero exhibit transient neurological, respiratory and gastrointestinal symptoms in the early postnatal period, which is commonly referred to as poor neonatal adaptation syndrome (PNAS). These manifestations may reflect either an abrupt discontinuation of drug exposure after birth or transient dysregulation of the neonatal serotonergic system [5,6,7,8].

Several neonatal outcomes have been associated with prenatal SSRI exposure, including slightly lower gestational age (GA), lower Apgar scores, and an increased risk of preterm delivery [9]. However, beyond these global outcomes, the impact of SSRIs on early physiological adaptation processes remains incompletely understood. SSRIs act through inhibition of the serotonin transporter (SERT), thereby increasing the extracellular serotonin levels. While serotonin is widely recognized for its role in central nervous system function [10,11], approximately 90% of the body’s serotonin is produced in the gastrointestinal tract by enterochromaffin cells and enteric neurons, where it plays a key role in regulating gut physiology [12,13].

Serotonin is critically involved in gastrointestinal motility, secretion, visceral sensitivity, epithelial homeostasis, and inflammatory responses [12,13]. Disruption of serotonergic signaling during fetal development may therefore influence the maturation and function of the enteric nervous system. Experimental and clinical evidence supports this hypothesis: in utero exposure to SSRIs has been associated with altered enteric nervous system development and increased gastrointestinal symptoms later in childhood [14,15]. Animal models further demonstrate that prenatal SSRI exposure can modify the gastrointestinal structure and function in offspring [16].

In parallel, serotonergic pathways also contribute to lower urinary tract function. Pharmacologic modulation of serotonin has been associated with urinary disturbances, including incontinence, and may influence bladder regulation [17,18]. These observations suggest that prenatal SSRI exposure could plausibly affect both gastrointestinal and urinary neonatal adaptation.

Despite this biological plausibility, the relationship between prenatal SSRI exposure and early neonatal elimination patterns has not been systematically studied. Time to first stool and time to first void are routinely monitored clinical parameters in the immediate postnatal period, reflecting gastrointestinal motility and renal–urinary adaptation. Deviations from expected timing may prompt further clinical evaluation; however, their association with in utero pharmacologic exposures remains poorly characterized.

Addressing this gap is particularly relevant, as early neonatal adaptation represents a critical transition period during which subtle functional alterations may be detectable, even in otherwise healthy neonates. Furthermore, intrapartum factors such as meconium-stained amniotic fluid (MSAF), which has been associated with both fetal stress and maternal SSRI use [19,20,21], may interact with these processes and contribute to variability in early elimination patterns.

Therefore, we aimed to evaluate whether maternal SSRI use during pregnancy is associated with time to first void and time to first stool in a cohort of healthy neonates. Given the central role of serotonin in gastrointestinal and urinary physiology, we hypothesized that prenatal SSRI exposure would be associated with altered neonatal elimination patterns.

## 2. Methods

### 2.1. Study Design and Participants

This retrospective matched cohort study was conducted at a tertiary referral center with approximately 12,000 deliveries annually. We reviewed electronic medical records of neonates admitted to the well-baby nursery between January 2015 and May 2019. Eligible participants were healthy singleton neonates born between 35 + 0 and 41 + 6 weeks of gestation, with a 5 min Apgar score of ≥8. The exposed group included neonates born to mothers who used SSRI drugs as monotherapy during pregnancy. SSRI exposure was defined based on documentation in maternal medical records at the time of delivery and recorded as a binary variable (yes/no). When available, the specific SSRI agent was documented; however, analyses were not stratified by agent due to sample size limitations. Exclusion criteria included multiple gestations, combined antidepressant therapy, oligohydramnios during pregnancy, congenital malformations of the urinary system, congenital heart defects, and neonatal intensive care unit admissions.

Each exposed neonate was matched 1:1 to an unexposed control, based on GA and size-for-gestational-age category (appropriate [AGA], small [SGA] and large [LGA]) [22]. When multiple eligible controls were available, the temporally closest birth was selected. The local institutional review board, the Helsinki committee, approved this study (0408-19-TLV) and waived the requirement for informed consent due to the retrospective design. The study was carried out in accordance with the Good Clinical Practice guidelines and the Declaration of Helsinki.

### 2.2. Data Collection

The maternal data included age, medical conditions, and medication use during pregnancy. The neonatal data included GA, gender, birth weight (BW), mode of delivery, and clinical parameters such as meconium-stained amniotic fluid (MSAF), crying after birth and respiratory distress, and feeding characteristics. Feeding variables included the time to initiation of enteral feeding and feeding regimen at discharge.

Primary outcomes were time to first void and time to first stool, recorded in hours after birth. Additional data included weight loss at discharge and the Finnegan Neonatal Abstinence Scoring System, a standardized tool used to assess neonatal withdrawal symptoms [23].

### 2.3. Statistical Analyses

Categorical variables are presented as counts and percentages, and continuous variables as mean ± standard deviation or median [interquartile range], as appropriate. Normality was assessed using Shapiro–Wilk tests, histograms and Q-Q plots. Between-group comparisons were performed using chi-square tests or Fisher’s exact tests for categorical variables and Student’s *t*-test or Mann–Whitney U test for continuous variables, as appropriate.

To assess the independent association prenatal SSRI exposure and time to first stool, a multivariable linear regression model was constructed. Due to right-skewed distribution, time to first stool was log10-transformed prior to analysis. Regression coefficients are reported on the log-transformed scale. The primary model was adjusted for maternal age, GA, and neonatal sex. BW was not included due to its collinearity with GA and matching on size-for-gestational-age. Sensitivity analyses were performed to assess the robustness of the findings: (1) inclusion of size-for-gestational-age category and (2) inclusion of time to first feeding. Model assumptions, including linearity, homoscedasticity, and absence of multicollinearity, were assessed.

All statistical tests were two-sided and *p* < 0.05 was considered statistically significant. Analyses were performed using IBM SPSS Statistics for Windows, version 29 (IBM Corp., Armonk, NY, USA).

## 3. Results

A total of 266 neonates were included, comprising 133 exposed to SSRIs in utero and 133 matched unexposed controls. The baseline perinatal and neonatal characteristics are presented in Table 1.

Mothers in the SSRI-exposed group were older than those in the control group (34.5 ± 4.8 vs. 32.2 ± 4.8 years, *p* < 0.001). As per the study design, the GA and weight-for-gestational-age distribution did not differ between groups. Other maternal and perinatal characteristics, including delivery mode and anesthesia, were similar.

The timing of the first void did not differ between the groups (median 10 [IQR 6.7–14.3] vs. 9.4 [6.2–13.3] hours, *p* = 0.460), nor did the proportion of neonates voiding after 24 h.

Neonates exposed to SSRIs had a shorter median time to first stool compared with unexposed neonates (7.4 [4.6–11.5] vs. 8.6 [6.0–12.8] hours, *p* = 0.023), although the absolute difference was modest.

The rate of MSAF was higher among SSRI-exposed neonates (24.1% vs. 16.5%), although this difference did not reach statistical significance (*p* = 0.127). Among SSRI-exposed neonates, those with MSAF had a significantly shorter time to first stool compared to those without MSAF (5.6 [3.7–8.4] vs. 8.3 [5.1–11.9] hours, *p* = 0.009). In contrast, among unexposed neonates, the time to first stool was numerically shorter in the presence of MSAF (7.3 [5.6–10.6] vs. 9.2 [6.7–13.1] hours), but this difference did not reach statistical significance (*p* = 0.068). Time to first void did not differ according to MSAF status in either group (Table 2).

Initiation of enteral feeding occurred at a similar time in both groups. However, feeding practices at discharge differed significantly (*p* = 0.001): neonates in the control group were more likely to be fed with their mother’s own milk (29.3% vs. 17.3%), whereas formula feeding was more common among SSRI-exposed neonates (19.5% vs. 6%).

Finnegan scores in the SSRI-exposed group were low (≤4) and were not associated with time to first void, time to first stool, initiation of feeding, weight loss at discharge, or clinical outcomes including MSAF, respiratory distress, or absence of crying at birth.

In multivariable linear regression using log10-transformed time to first stool, SSRI exposure was independently associated with a shorter time to first stool (B = −0.08, 95% CI −0.16 to −0.01, *p* = 0.035), corresponding to an approximate 17% reduction. GA, sex, and maternal age were not significantly associated with the outcome. The overall model explained a limited proportion of variance (R^2^ = 0.022). Sensitivity analyses yielded consistent results: inclusion of size-for-gestational-age or time to first feeding did not materially alter the association.

## 4. Discussion

In this retrospective matched cohort study of healthy neonates, prenatal SSRI exposure was associated with a modest but consistent reduction in time to first stool, while time to first void was unaffected. This association persisted after adjustment for relevant maternal and neonatal factors and remained robust across sensitivity analyses, supporting a small but independent association of in utero SSRI exposure on early neonatal gastrointestinal adaptation. On the log-transformed scale, this corresponds to an approximate 15–20% reduction in time to first stool. However, the clinical significance of this difference remains uncertain.

The observed association is biologically plausible, given the role of serotonin in gastrointestinal regulation. Serotonin is a key mediator within the enteric nervous system, where it regulates intestinal motility and peristaltic reflexes [11,12,13]. As SSRIs cross the placenta and influence serotonergic signaling [6,7], in utero exposure may affect neonatal gastrointestinal function. Experimental data further support the impact of the prenatal SSRI exposure on intestinal motility through modulation of serotonergic pathways [14,16]. In this context, the shorter time to first stool may reflect subtle alterations in early gut motility. Importantly, the association remained stable after accounting for fetal growth and early postnatal factors. Adjustment for size-for-gestational-age did not materially change the results, and the inclusion of time to first feeding in sensitivity analyses did not attenuate the association, arguing against these variables as primary drivers of the observed effect.

Feeding practices at discharge differed between groups, with lower rates of mother’s own milk and higher rates of formula feeding among SSRI-exposed neonates. This observation can be related to previous reports on the impact of SSRIs on the lactation process. Holland et al. have observed a reduced milk supply in six nursing mothers treated with sertraline [24]. Marshall et al.’s study of 431 women demonstrated that those on SSRI were more likely to experience delayed secretory activation [25], probably due to modulation of secretory activation in the metabolism of the mammary glands that are influenced by serotonin. As early feeding may influence gastrointestinal motility, this variable could potentially contribute to differences in time to first stool. However, given that adjustment for timing of feeding initiation did not alter the observed association, feeding-related factors are unlikely to fully explain the findings, although residual confounding cannot be excluded.

In addition, intrapartum factors such as MSAF, previously associated with maternal SSRI use [19,20] and fetal stress [21], were associated with shorter time to first stool among SSRI-exposed neonates. This association was not statistically significant in the unexposed group, suggesting that the relationship between MSAF and early stool passage may differ according to SSRI exposure. Although these findings raise the possibility of an interaction between serotonergic exposure and intrapartum factors, the study was not designed to formally test interaction effects, and these results should be interpreted cautiously. These findings suggest that the observed effect of SSRI exposure on stool timing may be influenced by intrapartum conditions, although confirmation would require formal interaction analysis in larger cohorts.

Despite the consistent association, the overall explanatory power of the models was limited and the global model fit was not statistically significant. This likely reflects the multifactorial nature of early neonatal stool passage, which is influenced by factors not captured in this dataset, including detailed feeding dynamics, perinatal stress, and early gut adaptation. Accordingly, these models should be interpreted as assessing association rather than prediction.

From a methodological perspective, variables potentially lying on the causal pathway, such as feeding-related factors, were excluded from the primary model and evaluated only in sensitivity analyses. MSAF was not included in the primary regression model, as it may represent an intrapartum factor or downstream manifestation of fetal processes rather than a baseline confounder, particularly given its known association with fetal stress and intrapartum conditions. Given its potential role in the causal pathway or as an effect modifier, it was instead explored in stratified analyses. In addition, given matching on GA and weight-for-gestational-age category, BW was not included in the primary model to avoid overlapping constructs of neonatal size and maturity.

This study has several limitations. Its retrospective design limits causal inference, and residual confounding, including confounding by indication, cannot be excluded. SSRI exposure was based on medical records and lacked detailed information on dose, duration, and timing. In addition, outcome measures relied on charted timing and may be subject to documentation variability. The modest effect size and low explained variance further suggest that important determinants of neonatal gastrointestinal adaptation were not captured.

In conclusion, prenatal SSRI exposure was associated with a modest but consistent reduction in time to first stool in otherwise healthy neonates, without affecting time to first void. While the clinical significance of this finding remains uncertain, the consistency of the association across analytic approaches, together with findings suggesting that intrapartum factors such as MSAF may modify the observed association, supports a potential influence of in utero SSRI exposure on early neonatal gastrointestinal function. Further prospective studies are needed to clarify underlying mechanisms and clinical implications.

## Figures and Tables

**Table 1:** Comparison of perinatal and neonatal data of neonates exposed to SSRIs in utero with neonates unexposed to SSRIs (control group).

Variable	Exposed to SSRI (*N* = 133)	Unexposed to SSRI (*N* = 133)	*p* Value
Maternal age	34.5 ± 4.8 (21–48)	32.2 ± 4.8 (20–45)	**<0.001**
Gestational diabetes mellitus	16 (12)	10 (7.5)	0.215
Maternal asthma	4 (3)	0	0.122
Maternal hypothyroidism	14 (10.5)	6 (4.5)	0.063
Maternal anemia	4 (3)	5 (3.8)	>0.99
Gestational age (week)	39 (38–39.5)	39 (38–39.5)	>0.99
Birth weight (g)	3167.9 ± 432.4 (2153–4380)	3189.4 ± 416.5 (2175–4165)	0.679
Female	76 (57.1)	68 (51.1)	0.325
Delivery mode			0.830
Vaginal	95 (71.4)	92 (69.2)	
Vacuum	11 (8.3)	10 (7.5)	
Caesarian section	27 (20.3)	31 (23.3)	
Anesthesia during delivery			0.738
None	30 (22.6)	26 (19.5)	
Epidural and spinal	102 (76.7)	105 (78.9)	
General	1 (0.8)	2 (1.5)	
Appropriate for gestational age	113 (85)	113 (85)	>0.99
Small for gestational age	11 (8.3)	11 (8.3)	
Large for gestational age	9 (6.8)	9 (6.8)	
Meconium-stained amniotic fluid	32 (24.1)	22 (16.5)	0.127
Time to first void (h)	10 (6.7–14.3)	9.4 (6.2–13.3)	0.460
First voiding above 24 h	5 (3.8)	3 (2.3)	0.722
Time to first stool (h)	7.4 (4.6–11.5)	8.6 (6–12.8)	0.023
Start of enteral feeding (h)	8 (4.3–10.8)	7.2 (3.3–10.3)	0.319
Feeding regimen at discharge			**0.001**
Mother’s own milk	23 (17.3)	39 (29.3)	
Formula	26 (19.5)	8 (6)	
Mixed	84 (63.2)	86 (64.7)	
Weight loss at discharge (%)	5.6 ± 2.1 (1.4–12.9)	5.7 ± 1.7 (1–9.5)	0.504
Not crying shortly after birth	9 (6.8)	5 (3.8)	0.272
Respiratory distress	34 (25.6)	30 (22.6)	0.566
Spitting	53 (39.8)	53 (39.8)	>0.99
Finnegan Neonatal Abstinence Scoring System		NA	
0	43 (33.6)		
1	75 (58.6)		
2	4 (3.1)		
3	4 (3.1)		
4	2 (1.6)		

Data are expressed by median [IQR], mean ± SD (range) or *n* (%); in bold, significant *p*.

**Table 2:** Time to first stool and time to first void according to meconium-stained amniotic fluid (MSAF) status, stratified by SSRI exposure.

	+SSRI			−SSRI		
	+MSAF	−MSAF	*p* Value	+MSAF	−MSAF	*p* Value
Time to first stool	5.6 [3.7, 8.4]	8.3 [5.1, 11.9]	**0.009**	7.3 [5.6, 10.6]	9.2 [6.7, 13.1]	0.068
Time to first void	11.3 [7.1, 15.8]	9.8 [6.5, 13.6]	0.240	9 [7.2, 13.1]	9.7 [6.1, 13.4]	0.858

Data are expressed by median [IQR]; in bold, significant *p*.

## Data Availability

All data generated or analyzed during this study are included in this article. For further inquiries, please contact the corresponding author.
